# Attitudes and barriers to breastfeeding among women at high-risk for not breastfeeding: a prospective observational study

**DOI:** 10.1186/s12884-024-06264-x

**Published:** 2024-01-24

**Authors:** Jessica Cole, Ateshi Bhatt, Andrew G. Chapple, Sarah Buzhardt, Elizabeth F. Sutton

**Affiliations:** 1 Department of Obstetrics and Gynecology, Louisiana State University Health and Sciences Center, Baton Rouge, LA 70817 USA; 2 Woman’s Hospital Research Center, Woman’s Hospital, Baton Rouge, LA 70817 USA; 3 Department of Interdisciplinary Oncology, School of Medicine, Louisiana State University Health and Sciences Center, New Orleans, LA 70112 USA

**Keywords:** Breastfeeding, Obstetrics, Prenatal care, Pregnancy

## Abstract

**Background:**

Rates of breastfeeding are lower among minority and underserved populations in the United States. Our study objective was to assess pregnant persons attitudes and barriers to breastfeeding among a cohort at high risk for not breastfeeding.

**Methods:**

We disseminated the Iowa Infant Feeding Attitude Scale (IIFAS) to 100 pregnant persons at least 18 years of age attending a prenatal visit in a low-resource, academic practice in south-central Louisiana (Woman’s Hospital). The IIFAS, as well as questions collecting information on breastfeeding experience and sociodemographic characteristics, were administered via interview. Medical records were reviewed to investigate associations between attitudes about breastfeeding in pregnancy and patient’s feeding choices during the delivery hospital stay. Fisher exact tests and Wilcoxon rank-sum tests were used to assess associations between categorical and continuous variables respectively.

**Results:**

Of the 98 participants who completed the study, 8% were Hispanic, 63% were Black, 95% were Medicaid eligible, and 50% were unemployed. 59% (*n* = 58) went on to breastfeed/combination breast-formula feed (called “Any-Breastfeeding Group”) during the delivery stay. Total IIFAS score during pregnancy was significantly higher among those who went on to breastfeed during delivery hospital stay (Any-Breastfeeding Group vs. Formula-Feeding-Only Group: 58.9 ± 5.5 vs. 53.7 ± 6.2 respectively, *p* < 0.001). In the group that went on to only formula feed (Formula-Feeding-Only Group), only 4% agreed breastfeeding was more convenient when surveyed during pregnancy, compared to 45% of the Any-Breastfeeding Group. 60% of Formula-Feeding-Only Group agreed formula is as healthy as breast milk.

**Conclusion:**

The three major themes that coincided with favorability toward breastfeeding in the study, and can be addressed during prenatal counseling, are: mother-infant bonding, convenience, and health benefits. By identifying attitudes and barriers to breastfeeding for patients during pregnancy who went on to not breastfeed, directed educational opportunities can be developed to address these specific attitudes to ultimately increase breastfeeding initiation and continuation.

## Background

Breastfeeding supports physiologic, metabolic, and emotional benefits for both breastfeeders and those breastfed [1, 2]. While the incidence of breastfeeding has steadily increased in the United States (US) since the start of the 21st century, disparities are seen among racial and ethnic minority birthing persons with lower rates of initiation of breastfeeding, lower rates of exclusive breastfeeding, and higher rates of breastfeeding cessation compared to non-Hispanic White persons [1, 3–6]. Barriers from literacy, articulation, employment, and acceptance and support towards breastfeeding cause negative perceptions of breastfeeding, especially among minority populations [1, 7]. While there has been an improvement in breastfeeding rates among Black birthing persons, this minority group continues to have the lowest rates of breastfeeding initiation in the US, with Black birthing persons 2.5 times less likely to breastfeed than White birthing persons [1].

Maternal intention to breastfeed and breastfeeding self-efficacy are well established predictors for breastfeeding uptake and duration [8, 9]. Evidence for the effectiveness of education for breastfeeding success is less consistent. A review by Wouk et al. reported prenatal education interventions increased breastfeeding uptake and duration [10], while another review by Lumbiganon et al. reported inconclusive evidence to support the effectiveness of education for initiation and duration of breastfeeding [11]. Findings that maternal intention increases breastfeeding uptake alongside evidence of inconsistent effectiveness for breastfeeding education leads to the question: can breastfeeding education be optimized and/or customized to increase breastfeeding uptake by encouraging maternal breastfeeding intention? The aim of this study was to assess attitudes and self-reported barriers to breastfeeding among pregnant women at high risk for not breastfeeding in a low-resource OB/GYN clinic. We hypothesized that a poor attitude towards breastfeeding in pregnancy is associated with a decreased likelihood of breastfeeding during hospital stay for delivery. To test our hypothesis, we conducted a prospective observational study disseminating the Iowa Infant Feeding Attitude Scale (IIFAS) survey [12] to 100 pregnant women at least 18 years of age attending a prenatal visit.

## Methods

### Study design

A prospective cohort study enrolled 100 pregnant women at least 18 years old receiving care at an OB/GYN clinic (Baton Rouge, Louisiana) between June and August 2021. A survey collecting sociodemographic variables (age, race, ethnicity, education, annual income, employment, and health insurance), breastfeeding experience, and attitudes towards breastfeeding using the Iowa Infant Feeding Attitude Scale (IIFAS) was administered by an interview between the study coordinator and participant at the study visit during pregnancy [12]. Participants’ breastfeeding initiation, breastfeeding status at discharge from delivery stay, as well as infant feeding plans recorded at time of survey and during hospital admission for delivery, were abstracted by chart review and compared to the IIFAS score and sociodemographic characteristics. Of note, the clinic within which the study was conducted universally provides general prenatal education materials (four handouts include breastfeeding information: Making an informed decision on breastfeeding; Fall in love- your first hour together; Learn your baby- strengthening your bond; Home sweet home- see how baby shows what s/he wants!) that include information regarding breastfeeding to all pregnant persons at their initial obstetric clinic visit. The primary outcome was any breastfeeding (i.e., exclusive or in combination with formula feeding) at time of discharge from delivery hospital stay. This study was approved and monitored by Woman’s Hospital Foundation Institutional Review Board (FWA00005699), and informed consent was obtained from participants prior to initiation of study procedures.

### Breastfeeding experience and exposure

Participants were asked questions via interview about their experiences and exposure to breastfeeding, including if they breastfed before (if applicable), if they knew anyone who breastfed, if they were breastfed as an infant, if their partner was supportive of breastfeeding (if applicable), if they have a place to pump breastmilk at their work if employed, and if they had remembered being provided information about breastfeeding in their prenatal care.

### Iowa infant feeding attitude scale

The Iowa Infant Feeding Attitude Scale (IIFAS) was administered at a single time point during pregnancy [12]. The scale is composed of 17 items with a 5-point Likert scale ranging from 1 (strongly disagree) to 5 (strongly agree). The total IIFAS score can range from 17 to 85, with a higher score indicating more a positive attitude toward breastfeeding [12]. The IIFAS is structured with favorable breastfeeding attitude items (#3, 5, 7, 9, 12, 13, 15, and 16; where “strongly agree” indicates a favorable attitude; Table 4) and unfavorable breastfeeding attitude items (#1, 2, 4, 6, 8, 10, 11, 14, and 17; where “strongly agree” indicates a less favorable attitude i.e., reverse scored; Table 5).

### Delivery record abstraction

Abstraction from the electronic medical record was completed after delivery. Feeding plan upon admission for delivery, breastfeeding initiation, lactation consultation, and feeding status at discharge were abstracted, and an oversample (20%) validated for accuracy by a second reviewer.

### Statistical analysis

Categorical variables were summarized by reporting counts and percentages while continuous variables were summarized by reporting means and standard deviations. Fisher exact tests were used to assess associations between categorical variables while Wilcoxon rank-sum tests were used to compare continuous variables across groups.

## Results

### Study population

100 participants were enrolled, and 98 participants completed the study. Two participants delivered outside the study facility and delivery records were not available. Among the diverse cohort, 63% (*n* = 62) were Black, 8% (*n* = 8) were Hispanic, the majority were Medicaid eligible (95%, *n* = 93) and 50% (*n* = 49) were receiving WIC benefits during pregnancy (Table 1). 60% of participants were between 18 and 25 years old (*n* = 59). Most reported a high school diploma (68%) or less than a high school diploma (14%) as the highest level of education completed. 50% (*n* = 49) were unemployed at time of enrollment and only 9% (*n* = 9) reported a salary of at least $50,000 per year. The majority (71%) of participants reported their pregnancy was not planned (Table 1).

**Table 1:** Population characteristics

	All (*n* = 98)	Any-Breastfeeders (*n* = 58)	Formula-Only Feeders (*n* = 40)	*P*-value	% breastfeeding at discharge
Age				0.845	
18–25 years old	59 (60.2%)	34 (58.6%)	25 (62.5%)		57.6
25–30 years old	20 (20.4%)	13 (22.4%)	7 (17.5%)		65.0
30 + years old	19 (19.4%)	11 (19%)	8 (20%)		57.9
Ethnicity				***0.020***	
Hispanic	8 (8.2%)	8 (13.8%)	0 (0%)		100
Non-Hispanic	90 (91.8%)	50 (86.2%)	40 (100%)		55.6
Race				***0.001***	
White, Other	36 (36.7%)	29 (50.0%)	7 (17.5%)		80.6
Black	62 (63.3%)	29 (50.0%)	33 (82.5%)		46.8
GA at time of IIFAS survey				0.132	
< 14 weeks	8 (8.2%)	2 (3.4%)	6 (15%)		25.0
14–27 weeks	28 (28.6%)	18 (31%)	10 (25%)		64.3
28 + weeks	62 (63.3%)	38 (65.5%)	24 (60%)		61.3
Education				0.522	
College Degree+	17 (17.3%)	12 (20.7%)	5 (12.5%)		70.6
High school degree	67 (68.4%)	39 (67.2%)	28 (70%)		58.2
Less than a high school diploma	14 (14.3%)	7 (12.1%)	7 (17.5%)		50.0
Income				0.206	
Under $10,000 USD per year	27 (27.6%)	12 (20.7%)	15 (37.5%)		44.4
$10,000-$25,000 per year	31 (31.6%)	18 (31%)	13 (32.5%)		58.1
$25,000-$50,000 per year	31 (31.6%)	21 (36.2%)	10 (25.0%)		67.7
$50k+	9 (9.2%)	7 (12.1%)	2 (5.0%)		77.8
Employment				0.014	
Full-time	34 (34.7%)	16 (27.6%)	18 (45.0%)		47.1
None	49 (50.0%)	36 (62.1%)	13 (32.5%)		73.5
Part-time	15 (15.3%)	6 (10.3%)	9 (22.5%)		40.0
Parity, mean ± sd	1.12 ± 1.29	1.26 ± 1.43	0.92 ± 1.02	0.387	
Planned pregnancy				0.172	
No	70 (71.4%)	38 (65.5%)	32 (80%)		54.3
Yes	28 (28.6%)	20 (34.5%)	8 (20%)		71.4
Health Insurance				0.787	
Medicaid	93 (94.9%)	54 (93.1%)	39 (97.5%)		58.1
None	2 (2%)	2 (3.4%)	0 (0.0%)		100
Private	3 (3.1%)	2 (3.4%)	1 (2.5%)		66.7
WIC				0.078	
I don’t know	1 (1%)	0 (0%)	1 (2.5%)		0
No	48 (49%)	33 (56.9%)	15 (37.5%)		68.8
Yes	49 (50%)	25 (43.1%)	24 (60.0%)		51.0
IIFAS Score	56.77 ± 6.29	58.86 ± 5.53	53.73 ± 6.15	**< 0.001**	

### Breastfeeders vs. formula-only feeders

At discharge from hospital delivery stay, 59% (n = 58) of participants were breastfeeding (exclusively or in combination with formula feeding) (“Any-Breastfeeders”), and 41% (n = 40) were exclusively formula feeding (“Formula-Only Feeders”) (Table 1). Among the Hispanic population that was surveyed, 100% were Any-Breastfeeders at discharge. A significantly larger proportion of Formula-Only Feeders were Black (83% of Formula-Only Feeders were Black vs. 50% of Any-Breastfeeders were Black, *p* = 0.001) and employed (45% of Formula-Only Feeders were employed vs. 28% of Any-Breastfeeders were employed, *p* = 0.014). A marginal increase in history of breastfeeding was seen in the Any-Breastfeeders compared to the Formula-Only Feeders (43% vs. 25% respectively, *p* = 0.087) (Table 2). When asked about previous exposure to breastfeeding, Any-Breastfeeders were significantly more likely to have been breastfed themselves compared to Formula-Only Feeders (40% vs. 12% respectively, *p* < 0.001). When asked about plans for infant feeding during pregnancy, 66 participants (67%) intended to breastfeed, and, of those, 79% were breastfeeding at time of discharge from hospital delivery stay (Table 2).

**Table 2:** Breastfeeding experience and environment

	All (*n* = 98)	Any-Breastfeeders (*n* = 58)	Formula-Only Feeders (*n* = 40)	*P*-value
**Breastfeeding Exposure**				
Breastfeeding History				0.087
No	63 (64%)	33 (57%)	30 (75%)	
Yes	35 (36%)	25 (43%)	10 (25%)	
Did your mom breastfeed you when you were a baby?				< 0.001
I don’t know	15 (15%)	12 (20%)	3 (8%)	
No	55 (56%)	23 (40%)	32 (80%)	
Yes	28 (29%)	23 (40%)	5 (12%)	
Do you know any family or friends who have ever breastfed their baby?				0.266
No	16 (16.3%)	7 (12.1%)	9 (22.5%)	
Yes	82 (83.7%)	51 (87.9%)	31 (77.5%)	
Do you feel like you would have time/place to pump breast milk at your place of employment?				0.259
No	27 (55%)	10 (46%)	17 (63%)	
Yes	22 (45%)	12 (54%)	10 (37%)	
If you have a partner, do they support you breastfeeding				0.098
I don’t have a partner	2 (2.0%)	1 (1.7%)	1 (2.5%)	
I don’t know	3 (3.1%)	0 (0%)	3 (7.5%)	
No	1 (1.0%)	1 (1.7%)	0 (0%)	
Yes	92 (93.9%)	56 (96.6%)	36 (90%)	
**Breastfeeding Education**				
Has your doctor or nurse ever talked to you about the health benefits of breastfeeding for moms and for their babies?				0.806
No	22 (22.4%)	14 (24.1%)	8 (20%)	
Yes	76 (77.6%)	44 (75.9%)	32 (80%)	
Have you been given any resources for learning about breastfeeding, like a handout or internet link?				0.629
No	23 (23.5%)	15 (25.9%)	8 (20%)	
Yes	75 (76.5%)	43 (74.1%)	32 (80%)	
**Feeding Plans**				
Have you decided how you are hoping to feed your baby?				< 0.001
Breastfeed only	40 (40.8%)	32 (55.2%)	8 (20%)	
Combination	26 (26.5%)	20 (34.5%)	6 (15%)	
Formula	24 (24.5%)	3 (5.2%)	21 (52.5%)	
None	8 (8.2%)	3 (5.2)	5 (12.5%)	
Feeding plan on admission for delivery				< 0.001
Breastfeeding	67 (68.4%)	58 (100%)	9 (22.5%)	
Formula feeding	30 (30.6%)	0 (0%)	30 (75%)	
Unknown/missing	1 (1%)	0 (0%)	1 (2.5%)	
**Infant Feeding Experience during Delivery Hospital Stay**				
Lactation visited patient during delivery stay				< 0.001
No	36 (36.7%)	1 (1.7%)	35 (87.5%)	
Yes	62 (63.3%)	57 (98.3%)	5 (12.5%)	
Any breastfeeding initiation? Baby to breast at least one time				< 0.001
No	37 (37.8%)	0 (0%)	37 (92.5%)	
Yes	61 (62.2%)	58 (100%)	3 (7.5%)	
Feeding status at discharge from delivery stay				< 0.001
Breastfeeding	23 (23.5%)	23 (39.7%)	0 (0%)	
Combination	35 (35.7%)	35 (60.3%)	0 (0%)	
Formula feeding	40 (40.8%)	0 (0%)	40 (100%)	

### Breastfeeding attitudes during pregnancy for participants breastfeeding vs. formula-feeding at delivery discharge

The average IIFAS score was 56.8 ± 6.3 (*n* = 98) when surveyed during pregnancy (Table 1). Total IIFAS score during pregnancy was moderately higher (indicating favorability to breastfeeding) among those who went on to breastfeed compared to participants who only formula-fed (Any-Breastfeeding Group vs. Formula-Feeding-Only Group: 58.9 ± 5.5 vs. 53.7 ± 6.2 respectively, *p* < 0.001) (Table 1). Comparing IIFAS scores to breastfeeding exposure history, participants who had previously breastfed had a marginally higher IIFAS (59.8±5.0 vs. 55.1±6.3, *p* < 0.001), while a personal history of having been breastfed themselves, knowing family or friends who have breastfed, having a supportive partner, or receiving breastfeeding education was not associated with significantly higher breastfeeding attitudes score (Table 3). Participants who reported in the study the intention to breastfeed as well as reported plans to breastfeed at hospital admission for delivery both have significantly higher IIFAS scores compared to those with formula-only feeding intentions (both *p* < 0.001; Table 3).

**Table 3:** IIFAS survey scores and breastfeeding exposure, education, and feeding plans

	Yes	No	*P*-value
**Breastfeeding Exposure**			
Breastfeeding history	59.8±5.0	55.1±6.3	< 0.001
Did your mom breastfeed you when you were a baby?	57.6±6.4	55.7±6.4	0.375
Do you know any family or friends who have ever breastfed their baby?	56.9±6.1	56±7.3	0.78
If you have a partner, do they support you breastfeeding?	57.0±6.2	59 (NA)	0.654
**Breastfeeding Education**			
Has your doctor or nurse ever talked to you about the health benefits of breastfeeding for moms and for their babies?	57.4±6.4	54.6±5.4	0.105
Have you been given any resources for learning about breastfeeding, like a handout or internet link?	57.1±6.2	55.7±6.5	0.718
	**Any Breastfeeding**	**Formula** **only**	***P***-**value**
**Feeding Plans**			
Have you decided how you are hoping to feed your baby?	58.7±5.5	52.5±6.5	< 0.001
Feeding plan on admission for delivery	59.0±5.4	51.9±5.5	< 0.001

Among the favorable attitude IIFAS items, item scores among Any-Breastfeeders were significantly higher for “breastfeeding increases mother-infant bonding”, “formula-fed babies are more likely to be overfed than are breast-fed babies”, “breast-feeding is more convenient than formula-feeding “, and “mothers who formula-feed miss one of the great joys of motherhood” compared to Formula-Only Feeders (all *p* < 0.05, Table 4).

**Table 4:** IIFAS survey scores for favorable breastfeeding items (counts (%) for each score by item)

	All (98)	Breastfeeding at discharge (*n* = 58)	Not breastfeeding at discharge (*n* = 40)	*P*-value
Breastfeeding increases mother-infant bonding.				***0.006***
Disagree	7 (7.1)	3 (5.2)	4 (10)	
Neutral	8 (8.2)	1 (1.7)	7 (17.5)	
Agree	56 (57.1)	33 (56.9)	23 (57.5)	
Strongly Agree	27 (27.6)	21 (36.2)	6 (15)	
Formula-fed babies are more likely to be overfed than are breast-fed babies.				***0.007***
Strongly disagree	4 (4.1)	3 (5.2)	1 (2.5)	
Disagree	34 (34.7)	12 (20.7)	22 (55)	
Neutral	26 (26.5)	20 (34.5)	6 (15)	
Agree	31 (31.6)	21 (36.2)	10 (25)	
Strongly Agree	3 (3.1)	2 (3.4)	1 (2.5)	
Mothers who formula-feed miss one of the great joys of motherhood.				***0.028***
Strongly disagree	5 (5.1)	1 (1.7)	4 (10)	
Disagree	46 (46.9)	23 (39.7)	23 (57.5)	
Neutral	13 (13.3)	8 (13.8)	5 (12.5)	
Agree	32 (32.7)	25 (43.1)	7 (17.5)	
Strongly Agree	2 (2)	1 (1.7)	1 (2.5)	
Babies fed breast milk are healthier than babies that are fed formula.				0.409
Strongly disagree	2 (2)	0 (0)	2 (5)	
Disagree	24 (24.5)	13 (22.4)	11 (27.5)	
Neutral	18 (18.4)	11 (19)	7 (17.5)	
Agree	42 (42.9)	25 (43.1)	17 (42.5)	
Strongly Agree	12 (12.2)	9 (15.5)	3 (7.5)	
Breast milk is the ideal food for babies.				0.340
Disagree	15 (15.3)	7 (12.1)	8 (20)	
Neutral	12 (12.2)	5 (8.6)	7 (17.5)	
Agree	59 (60.2)	38 (65.5)	21 (52.5)	
Strongly Agree	12 (12.2)	8 (13.8)	4 (10)	
Breast milk is more easily digested than formula.				0.427
Strongly disagree	1 (1)	0 (0)	1 (2.5)	
Disagree	9 (9.2)	4 (6.9)	5 (12.5)	
Neutral	30 (30.6)	17 (29.3)	13 (32.5)	
Agree	52 (53.1)	32 (55.2)	20 (50)	
Strongly Agree	6 (6.1)	5 (8.6)	1 (2.5)	
Breast-feeding is more convenient than formula feeding.				***0.010***
Disagree	33 (33.7)	13 (22.4)	20 (50)	
Neutral	25 (25.5)	14 (24.1)	11 (27.5)	
Agree	35 (35.7)	27 (46.6)	8 (20)	
Strongly Agree	5 (5.1)	4 (6.9)	1 (2.5)	

Among the reverse-scored, unfavorable attitude IIFAS items, only two were significantly different between Formula-Only Feeders and Any-Breastfeeders. Formula-Only Feeders agreed more that “women should not breastfeed in public places such as restaurants” compared to Any-Breastfeeders (*p* = 0.025, Table 5). Formula-Only Feeders also agreed that “formula is as healthy for an infant as breast milk” significantly more than Any-Breastfeeders (*p* = 0.035, Table 5).

**Table 5:** IIFAS survey scores for unfavorable breastfeeding items (counts (%) for each score by item)

	All (98)	Breastfeeding at discharge (*n* = 58)	Not breastfeeding at discharge (*n* = 40)	*P*-value
The nutritional benefits of breast milk last only until the baby is weaned from breast milk (meaning stops getting breast milk).				0.724
Strongly Agree	5 (5.1)	3 (5.2)	2 (5)	
Agree	25 (25.5)	14 (24.1)	11 (27.5)	
Neutral	34 (34.7)	18 (31)	16 (40)	
Disagree	32 (32.7)	22 (37.9)	10 (25)	
Strongly disagree	2 (2)	1 (1.7)	1 (2.5)	
Formula-feeding is more convenient than breastfeeding.				0.199
Strongly Agree	7 (7.1)	2 (3.4)	5 (12.5)	
Agree	31 (31.6)	16 (27.6)	15 (37.5)	
Neutral	13 (13.3)	7 (12.1)	6 (15)	
Disagree	42 (42.9)	29 (50)	13 (32.5)	
Strongly disagree	5 (5.1)	4 (6.9)	1 (2.5)	
Breast milk is lacking in iron.				0.321
Strongly Agree	2 (2)	0 (0)	2 (5)	
Agree	8 (8.2)	5 (8.6)	3 (7.5)	
Neutral	38 (38.8)	26 (44.8)	12 (30)	
Disagree	46 (46.9)	25 (43.1)	21 (52.5)	
Strongly disagree	4 (4.1)	2 (3.4)	2 (5)	
Formula-feeding is the better choice if a mother plans to work outside the home.				0.700
Strongly Agree	1 (1)	0 (0)	1 (2.5)	
Agree	37 (37.8)	21 (36.2)	16 (40)	
Neutral	21 (21.4)	12 (20.7)	9 (22.5)	
Disagree	35 (35.7)	23 (39.7)	12 (30)	
Strongly disagree	4 (4.1)	2 (3.4)	2 (5)	
Women should not breast-feed in public places such as restaurants.				***0.025***
Strongly Agree	1 (1)	0 (0)	1 (2.5)	
Agree	11 (11.2)	2 (3.4)	9 (22.5)	
Neutral	12 (12.2)	8 (13.8)	4 (10)	
Disagree	58 (59.2)	37 (63.8)	21 (52.5)	
Strongly disagree	16 (16.3)	11 (19)	5 (12.5)	
Breast-fed babies are more likely to be overfed than formula-fed babies.				0.634
Agree	10 (10.2)	5 (8.6)	5 (12.5)	
Neutral	27 (27.6)	18 (31)	9 (22.5)	
Disagree	58 (59.2)	34 (58.6)	24 (60)	
Strongly disagree	3 (3.1)	1 (1.7)	2 (5)	
Fathers feel left out if a mother breast-feeds.				0.383
Agree	8 (8.2)	5 (8.6)	3 (7.5)	
Neutral	8 (8.2)	7 (12.1)	1 (2.5)	
Disagree	76 (77.6)	43 (74.1)	33 (82.5)	
Strongly disagree	6 (6.1)	3 (5.2)	3 (7.5)	
Formula is as healthy for an infant as breast milk.				***0.035***
Agree	34 (34.7)	15 (25.9)	19 (47.5)	
Neutral	24 (24.5)	13 (22.4)	11 (27.5)	
Disagree	35 (35.7)	27 (46.6)	8 (20)	
Strongly disagree	5 (5.1)	3 (5.2)	2 (5)	
A mother who occasionally (sometimes) drinks alcohol should not breast-feed her baby.				0.090
Strongly Agree	18 (18.4)	7 (12.1)	11 (27.5)	
Agree	57 (58.2)	33 (56.9)	24 (60)	
Neutral	7 (7.1)	6 (10.3)	1 (2.5)	
Disagree	16 (16.3)	12 (20.7)	4 (10)	

## Discussion

We conducted a prospective observational study among a pregnant population at high risk for not breastfeeding based on race and socioeconomic status. Among a diverse cohort with approximately one-quarter exclusively breastfeeding and another one-third combination feeding at discharge, we observed moderate-to-high favorable attitudes towards breastfeeding, as well as identified differences in attitudes about breastfeeding during pregnancy between the Formula-Only Feeders and Any-Breastfeeders. Birthing persons were most likely to breastfeed if they were breastfed themselves, had plans while pregnant to attempt breastfeeding once baby was born, and had a more positive attitude towards breastfeeding (indicated by the IIFAS) during pregnancy.

The rational of this study was to explore if a concentrated population of disadvantage pregnant individuals shares themes in breastfeeding attitudes which could then inform breastfeeding education content to improve breastfeeding uptake. While there is conflicting evidence for the effectiveness of education for breastfeeding uptake and duration [10, 11, 13], the predictors of breastfeeding uptake are also variable and often dependent on the population studied [14–17]. This logically points to opportunities to customize education for improved relevancy and effectiveness.

Within our study, a significant portion of the cohort was minority race and/or ethnicity and lower socioeconomic status. Our study revealed themes of breastfeeding exposure and experience when comparing the Any-Breastfeeders to Formula-Only Feeders both common and not among the existing literature. Our study found increased favorability scores among Any-Breastfeeders, which has been previously reported [18]. The Any-Breastfeeders group had more favorable attitudes about breastfeeding mother-infant bonding with breastfeeding, health benefits for the infant with breastfeeding, and the overall convenience of breastfeeding. Interestingly, while partner support of breastfeeding is a common theme supporting breastfeeding uptake in the literature, there was no observed difference in partner support between Any-Breastfeeders and Formula-Only Feeders in this study [12]. The majority of Formula-Only Feeders didn’t feel like they had a place to breast pump at work, which may have contributed to a more unfavorable attitude toward breastfeeding. There was no significant difference between groups when asked if they had received education on breastfeeding, and no difference was observed in IIFAS score with prenatal breastfeeding education. Lack of effectiveness of breastfeeding education/orientation by healthcare providers to increase IIFAS score has been previously reported [19]. There was also no difference in parity between the feeding groups, but there was a marginal increase in favorable attitudes towards breastfeeding among those with a history of breastfeeding. A positive relationship between breastfeeding experience and breastfeeding attitudes has also been reported previously [20].

Our study revealed themes of favorable and unfavorable attitudes towards breastfeeding, which in turn, inform targetable counseling opportunities during pregnancy. The three major themes that coincided with favorability toward breastfeeding in the study that could be addressed during prenatal counseling were: mother-infant bonding, convenience, and health benefits. Focused counseling on the health benefits of breastfeeding for both infant and mother towards women with unfavorable breastfeeding attitudes may improve rates of breastfeeding. Moreover, since a large percentage of formula-feeders felt that there was not a location to pump milk at their work, ensuring that workplaces have pumping areas as well as education and resources for legal rights for a breastfeeding-working person could improve the attitude and convenience for the working breastfeeders. Lastly, normalizing breastfeeding and pumping in a social setting such as a restaurant may also improve the convenience and comfortability of breastfeeding for the breastfeeder.

Our study had several strengths and limitations. A strength of our study was the inclusion of a diverse cohort at high risk of not breastfeeding. We had a high percentage of Black persons and persons eligible for Medicaid. Additionally, we used a validated instrument (Iowa Infant Feeding Attitude Scale) to assess the attitudes toward breastfeeding in a prospective design, which further strengthened our study. However, it is important to note this was a single institution study employing convenience sampling with a smaller population size. Unfortunately, our Hispanic population was underrepresented likely because we only conducted our survey in English. Especially in the presence of a 100% breastfeeding rate demonstrated in this survey in our Hispanic population, future studies would be informative for this population. Another limitation of our study is lack of documentation for acceptability rate for enrollment. Participants were approached in an OB/GYN clinic and offered to participate in the study, however, the total number approached who declined was not recorded. Finally, per hospital policy, inpatient lactation services are provided to patients who express interest in breastfeeding during the hospital stay and therefore, there is an obvious bias for those who received breastfeeding support during their hospital stay (i.e., those who expressed interest in breastfeeding).

## Conclusion

In summary, among a cohort at high risk for not breastfeeding, overall attitudes about breastfeeding were favorable during pregnancy. However, breastfeeding education was not associated with breastfeeding favorability or breastfeeding uptake. By identifying attitudes and barriers to breastfeeding for patients during pregnancy who went on to not breastfeed, our goal was to identify directed, educational opportunities to specifically address these attitudes. With more insight regarding barriers to breastfeeding, there is promise that prenatal breastfeeding education could be customized to target these feelings to improve the likelihood of breastfeeding. Future research to implement this focused counseling during pregnancy and the effects of breastfeeding rates is warranted.

## Data Availability

The datasets used and analyzed during the current study are available from the corresponding author on reasonable request.

## References

[1] Jones KM, Power ML, Queenan JT, Schulkin J (2015). Racial and ethnic disparities in Breastfeeding. Breastfeed Med.

[2] Eidelman AI, Schanler RJ. Breastfeeding and the use of human milk. Pediatrics [Internet]. 2012 Mar [cited 2022 Dec 28];129(3). Available from: https://pubmed.ncbi.nlm.nih.gov/22371471/.

[3] Gallo S, Kogan K, Kitsantas P. Racial and Ethnic Differences in Reasons for Breastfeeding Cessation Among Women Participating in the Special Supplemental Nutrition Program for Women, Infants, and Children. J Midwifery Womens Health [Internet]. 2019 Nov 1 [cited 2023 Apr 19];64(6):725–33. Available from: https://pubmed.ncbi.nlm.nih.gov/31469235/. 10.1111/jmwh.13031 31469235

[4] Quintero SM, Strassle PD, Londoño Tobón A, Ponce S, Alhomsi A, Maldonado AI (2023). Race/ethnicity-specific associations between breastfeeding information source and breastfeeding rates among U.S. women. BMC Public Health.

[5] Chapman DJ, Pérez-Escamilla R (2012). Breastfeeding among minority women: moving from risk factors to interventions. Adv Nutr.

[6] Lactation I, of M (US.) C on NSDP and. Who Breastfeeds in the United States? 1991 [cited 2023 Apr 19]; Available from: https://www.ncbi.nlm.nih.gov/books/NBK235588/.

[7] (US) O of the SG, (US) C for DC and P, US) O on WH., (The Surgeon General’s Call to Action to Support Breastfeeding. Surg Gen Call to Action to Support Breastfeed [Internet]. 2011 [cited 2023 Dec 1]; Available from: https://www.ncbi.nlm.nih.gov/books/NBK52682/.

[8] Lau CYK, Lok KYW, Tarrant M. Breastfeeding Duration and the Theory of Planned Behavior and Breastfeeding Self-Efficacy Framework: A Systematic Review of Observational Studies. Matern Child Health J [Internet]. 2018 Mar 1 [cited 2023 Nov 30];22(3):327–42. Available from: https://pubmed.ncbi.nlm.nih.gov/29427014/. 10.1007/s10995-018-2453-x 29427014

[9] Lemoine FV, Witt C, Howard S, Chapple A, Pam LK, Sutton EF. Factors Attributed to Breastfeeding Success in a Tertiary Obstetric Hospital. Women’s Heal reports (New Rochelle, NY) [Internet]. 2022 Jul 1 [cited 2023 Jul 4];3(1):624–31. Available from: https://pubmed.ncbi.nlm.nih.gov/36185071/. 10.1089/whr.2022.0045 PMC9518802 36185071

[10] Wouk K, Tully KP, Labbok MH. Systematic Review of Evidence for Baby-Friendly Hospital Initiative Step 3. J Hum Lact [Internet]. 2017 Feb 1 [cited 2023 Nov 30];33(1):50–82. Available from: https://pubmed.ncbi.nlm.nih.gov/28135481/. 10.1177/0890334416679618 28135481

[11] Lumbiganon P, Martis R, Laopaiboon M, Festin MR, Ho JJ, Hakimi M. Antenatal breastfeeding education for increasing breastfeeding duration. Cochrane database Syst Rev [Internet]. 2016 Dec 6 [cited 2023 Nov 15];12(12). Available from: https://pubmed.ncbi.nlm.nih.gov/27922724/. 10.1002/14651858.CD006425.pub4 PMC6463791 27922724

[12] De La Mora A, Russell DW, Dungy CI, Losch M, Dusdieker L. The Iowa Infant Feeding Attitude Scale: Analysis of Reliability and Validity1. J Appl Soc Psychol [Internet]. 1999 Nov 1 [cited 2023 Mar 23];29(11):2362–80. Available from: https://onlinelibrary.wiley.com/doi/full/10.1111/j.1559-1816.1999.tb00115.x.

[13] Kehinde J, O’Donnell C, Grealish A (2023). The effectiveness of prenatal breastfeeding education on breastfeeding uptake postpartum: a systematic review. Midwifery.

[14] Scott JA, Binns CW, Oddy WH, Graham KI. Predictors of breastfeeding duration: evidence from a cohort study. Pediatrics [Internet]. 2006 [cited 2023 Dec 4];117(4). Available from: https://pubmed.ncbi.nlm.nih.gov/16585281/. 10.1542/peds.2005-1991 16585281

[15] Johnson A, Kirk R, Rosenblum KL, Muzik M. Enhancing breastfeeding rates among African American women: a systematic review of current psychosocial interventions. Breastfeed Med [Internet]. 2015 Feb 1 [cited 2023 Dec 4];10(1):45–62. Available from: https://pubmed.ncbi.nlm.nih.gov/25423601/. 10.1089/bfm.2014.0023 PMC4307211 25423601

[16] DeVane-Johnson S, Woods-Giscombé C, Thoyre S, Fogel C, Williams R. Integrative Literature Review of Factors Related to Breastfeeding in African American Women: Evidence for a Potential Paradigm Shift. J Hum Lact [Internet]. 2017 May 1 [cited 2023 Dec 4];33(2):435–47. Available from: https://pubmed.ncbi.nlm.nih.gov/28380305/. 10.1177/0890334417693209 28380305

[17] Bai Y, Wunderlich SM, Fly AD. Predicting intentions to continue exclusive breastfeeding for 6 months: a comparison among racial/ethnic groups. Matern Child Health J [Internet]. 2011 Nov [cited 2023 Dec 4];15(8):1257–64. Available from: https://pubmed.ncbi.nlm.nih.gov/21057864/. 10.1007/s10995-010-0703-7 21057864

[18] Twells LK, Midodzi WK, Ludlow V, Murphy-Goodridge J, Burrage L, Gill N et al. Assessing Infant Feeding Attitudes of Expectant Women in a Provincial Population in Canada: Validation of the Iowa Infant Feeding Attitude Scale. J Hum Lact [Internet]. 2016 Aug 1 [cited 2023 Dec 4];32(3):NP9–18. Available from: https://pubmed.ncbi.nlm.nih.gov/25425631/. 10.1177/0890334414559647 25425631

[19] Giugliani ERj, Caiaffa WT, Vogelhut J, Witter FR, Perman JA. Effect of breastfeeding support from different sources on mothers’ decisions to breastfeed. J Hum Lact [Internet]. 1994 [cited 2023 Dec 4];10(3):157–61. Available from: https://pubmed.ncbi.nlm.nih.gov/7619265/. 10.1177/089033449401000310 7619265

[20] Kloeblen-Tarver AS, Thompson NJ, Miner KR. Intent to breast-feed: the impact of attitudes, norms, parity, and experience. Am J Health Behav [Internet]. 2002 [cited 2023 Dec 4];26(3):182–7. Available from: https://pubmed.ncbi.nlm.nih.gov/12018754/. 10.5993/ajhb.26.3.3 12018754

